# Animal culture research should include avian nest construction

**DOI:** 10.1098/rsbl.2021.0327

**Published:** 2021-07-14

**Authors:** Alexis J. Breen

**Affiliations:** Department of Human Behavior, Ecology and Culture, Max Planck Institute for Evolutionary Anthropology, Leipzig 04103, Germany

**Keywords:** material culture, nest-construction culture, nesting traditions, nest building, social learning, zebra finch

## Abstract

Material culture—that is, group-shared and socially learned object-related behaviour(s)—is a widespread and diverse phenomenon in humans. For decades, researchers have sought to confirm the existence of material culture in non-human animals; however, the main study systems of interest—namely, tool making and/or using non-human primates and corvids—cannot provide such confirmatory evidence: because long-standing ethical and logistical constraints handicap the collection of necessary experimental data. Synthesizing evidence across decades and disciplines, here, I present a novel framework for (mechanistic, developmental, behavioural, and comparative) study on animal material culture: avian nest construction.

## 1. Introduction

In one of his many influential essays on the natural world, polymath Alfred Russel Wallace concluded that, just like humans, nest-building birds exhibit socially learned architectural ‘customs’ [1, p. 235], a phenomenon that contemporary scientists would classify as material culture. Despite this early, and later, similar claims [2–5], the hypothesis that material culture plays a role in avian nest construction remains virtually untested [6,7], probably owing, in part, to the now defunct belief (e.g. [8,9]) that birds build nests based solely on instinct; in spite of approximately 150 years of data to show that birds can learn from their own and others' nest-construction experience [10].

Nevertheless, animal material culture research has focused largely on putative cases of tool-use traditions in wild non-human primates and corvids (recent reviews: [11–14]). While inarguably informative, these kinds of field studies cannot clearly isolate cultural explanations for existing technological diversity from ecological and/or genetic ones [15,16]. Instead, the use of experimental methods such as cross-fostering (exchanging offspring between different groups of unrelated adults) would be a necessary first step towards ‘disentangling’ this nature versus nurture debate—an approach that is currently considered unethical in these study systems to apply *in situ* because candidate cultural traits could be altered or lost. Thus, experimental evidence of material culture in animals is lacking, although cultural explanations seem to support a burgeoning number of other types of shared behaviours (e.g. song, mate choice, and foraging decisions; as recently reviewed in [17]).

Here, inspired by Wallace [1], I argue that avian nest construction offers a promising new framework for research into animal material culture, by: firstly, synthesizing the available evidence; secondly, proposing two hypothetical experiments that use state-of-the-art methodologies; thirdly, suggesting one potential model ‘cultural’ system for such study; and finally, highlighting how the biology of breeding birds allows animal culture researchers to progress the current experimental landscape. Together, these points produce the view that nest-building birds stand to advance the fields of animal behaviour and animal culture research.

## 2. Evidence of nesting traditions

To build a case for material culture in avian nest construction, consider the nest-building behaviour of Corsican blue tits (*Cyanistes caeruleus*): spatio-temporal analyses show that the types of nest material used by females (the builders in this species) are consistent within, but differ between, study plots; that these community differences in females' nest-material use are repeatable across breeding seasons; and that differences in plant availability cannot sufficiently explain females’ nest-material selection [3]. Such behavioural variation might, then, be the joint product of so-called ‘cultural founder effects’ [18] and ‘social learning strategies’ [19], where local nesting traditions were generated by, in turn, the nest-material preference of the first female in each area to build, the neighbours that readily copied her, those that copied the copiers, and so on. This supposition is strengthened by recent data showing that: (i) during nest construction, female blue tits will visit—up to approximately 40 times—the started or completed nests of other breeding blue tits [20]; and (ii) nest composition in female blue tits, in terms of the proportion of feathers they use, is a weakly heritable trait (heritability estimate of 13%; [21]).

Persistent species-atypical group patterns in birds' nest-site selection—for example, nesting in a shrub rather than on the ground [5], or on land rather than over water [2]—are also candidates for behaviour shaped by cultural processes, such as cultural inheritance [22]. Indeed, this observed group specificity in how birds begin nest construction is comparable to the documented group specificity (considered cultural) in how chimpanzees begin termite ‘fishing’: prior to probing an underground termite nest with a stick, members of one group lean on their elbow, whereas members of another group lay on their side [23]. And cross-fostering data show that early-life experience, rather than genes, can play a dominant role in shaping first-time settlement decisions in breeding birds [24]. But arguably the most compelling data supporting a role for material culture in avian nest construction come from a decade-long study [4] on backyard birds in North America, as detailed below.

**Figure 1.**
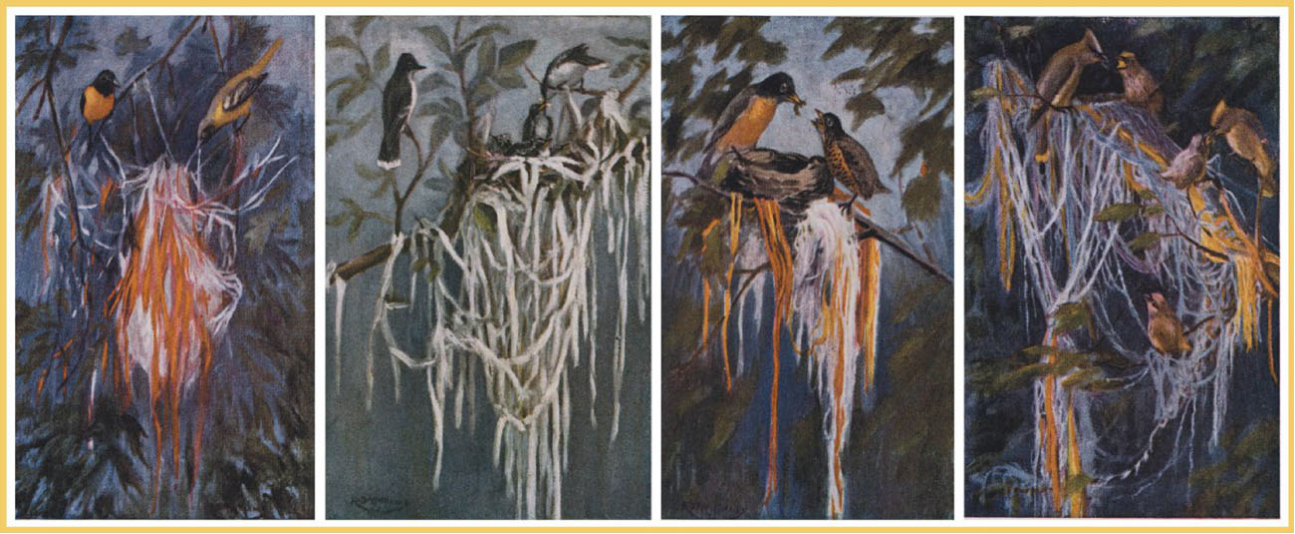
Backyard bird study. Paintings by naturalist Henry Smith Williams (1863–1943) depicting his decade-long observational study, wherein seven species, such as (left to right) orioles, flycatchers, robins, and waxwings, adopted the use of coloured strands of yarn for nest construction. Reproduced with permission by *Natural History*, November 1934 Copyright © Natural History Magazine, Inc., 1934.

In spring of 1923, lawyer, physician, naturalist and artist (figure 1) Henry Smith Williams [4] hung strands of red, white, blue, lavender, orange, and yellow yarn on a pole in his backyard; he did this every springtime up to 1932. His aim: to document which birds, if any, used which colour(s) of yarn for nest construction. From the start of the study, a female oriole (*Icterus* spp.) constructed her nest using pieces of each colour of yarn excluding the blue yarn. In the next years, other female orioles, too, followed suit, each with their own apparent material-colour preference(s). For example, in 1929, and again in 1930, one nest-building female oriole used nothing but white yarn. When, in 1931, the *entire* oriole colony (11 breeding pairs) preferred white yarn for nest construction, Williams credited this female with inspiring the ‘fashion of the season’. A similar claim could be made for the female kingbird (*Tyrannus* spp.) that, in 1929, constructed her nest exclusively from white yarn; she did this only after the orioles were finished constructing their respective nests. In subsequent seasons, two other kingbirds adopted the material-use behaviour, although, unlike the orioles, neither preferred the white yarn. In 1931, a preference for using one or multiple material colour(s) for nest construction was observed by Williams in an additional five species: American robins (*Turdus migratorius*), catbirds (*Ailuroedus* spp.), cedar waxwings (*Bombycilla cedrorum*), redstarts (*Phoenicurus* spp.), and least flycatchers (*Empidonax minimus*). Together, Williams' observational data coupled with his paintings (figure 1), provide tantalizing evidence of within and between-species social transmission of material preference in nest-building birds, spanning at least one, and up to 10 generations.

## 3. Methods for studying nesting traditions

**Figure 2.**
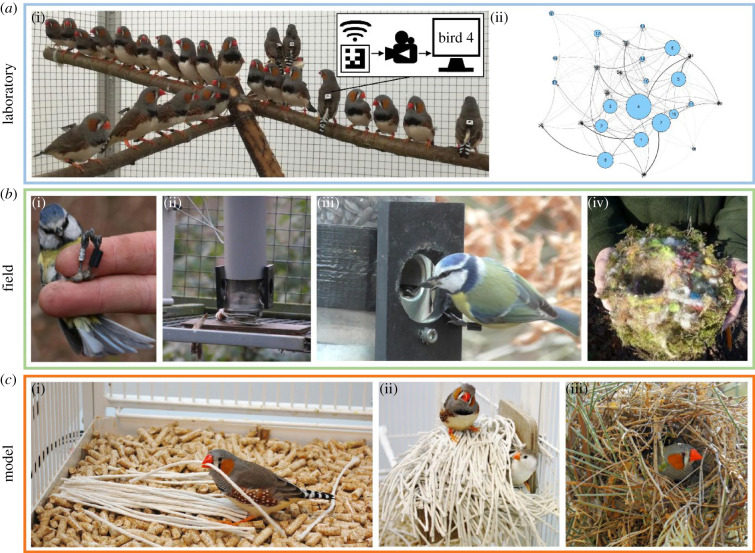
Methods and model. (*a*) Laboratory tracking technology: (i) laboratory-housed zebra finches wearing barcode-labelled backpacks that computer software can simultaneously track from video and/or photo recordings to reveal (ii) within-group social networks such as dominance hierarchies—for example, in this simulated network bird 4 displaced the most conspecifics and is thus the most dominant bird (i.e. has the biggest circle). (*b*) Field tracking technology: (i) a blue tit fitted with a (black) leg ring PIT tag that can communicate with (ii) a wired feeding station to (iii) timestamp individual feeding events; these stations could be modified to offer and record the choice of coloured nest material, (iv) which nest-building birds will use. (*c*) Zebra finches present one potential model ‘cultural’ system, as their nest construction can be studied experimentally under both (i,ii) laboratory and (iii) field conditions. Image credit: top panel, (i) Adriana Maldonado-Chaparro; middle panel, (i–iii) Friederike Hillemann and (vi) Michael Saynor; bottom panel, (i, ii) Eira Ihalainen and (iii) Chris Bellette.

The observational data synthesized above, however suggestive, cannot confirm the existence of nesting traditions in birds. In addition to established experimental methods such as cross-fostering, more recent empirical approaches plus newly developed animal behaviour tracking tools (figure 2) could be used to help fill this knowledge gap. Two hypothetical experiments are outlined below to illustrate the potential application of some of these methodologies to the study of material culture in avian nest construction (for in-depth reviews on animal behaviour tracking technology, see [25,26]).

In the first imagined experiment, the question being asked is if nesting cultures can establish in birds living in, for example, indoor and adjacent aviaries. In one aviary, birds build nests with one colour of material (e.g. pink string), while, in an adjacent aviary, birds with no experience of nest construction look on. Given that material-colour preference can be socially transmitted between pairs of builder–observer birds under laboratory conditions [27], it seems plausible that, when given pink and, say, orange string, the group of novice builder-birds could conform to the perceived local nest-construction culture—that is, construct nests mostly, if not entirely of pink string. It also seems plausible that within-group social dynamics (e.g. age, rank and/or relatedness) might differentially influence the rate of any such cultural transmission of information [19,28,29]. Dominant (i.e. more aggressive) individuals may, for example, adopt the apparent nesting tradition more quickly than subordinate individuals by chasing subordinates away from, and consequently, monopolizing access to, nest material [30]. In recent years, network-based diffusion analysis has become the go-to method for testing predictions about the spread of behaviour (for a step-by-step overview, see [31]). Critically, this method requires *a priori* knowledge of the strength of within-group member-to-member associations—in other words, how ‘friendly’ group members are with one another, from not at all to very. Cutting-edge backpack tracking tags (non-invasive harnesses mounted with unique, camera-detectable and software-readable barcodes that encode bird identity, position, and direction; figure 2*a*) should facilitate such social network mapping of the birds in the observer group in this make-believe scenario [32–34]; their dominance hierarchies might be mapped, for instance, using agonistic interactions—e.g. dyadic displacement data—extracted from barcodes detected at a filmed feeding period prior to nest construction (*sensu* [33]; figure 2*a*). Thus, the data generated from the first imagined experiment could reveal nest-building birds' capacity for, and candidate social processes involved in, animal material culture.

In the second imagined experiment, the question of the potential for nesting cultures remains the same but the study setting changes—the birds are now living freely in the wild. Recent research using passive integrated transponder (PIT) tags in combination with tracking feeding stations (e.g. [35–38]) suggests that such a change in the experimental environment is logistically manageable: because together these studies show that the identity and the foraging behaviour of wild target birds (that is, those with a PIT-tag equipped leg ring) can be catalogued automatically (figure 2*b*). Rather than offer food, then, these tracking stations could be adapted to offer a choice of at least two colours of nest material, such as dyed wool strands, cushion stuffing, or feathers—material types which are used by a number of builder-birds for nest construction [39,40] (figure 2*b*). Thus, data on who used which coloured material, and when, would be generated from this final make-believe scenario, providing insight into whether, and if so, how nesting traditions in wild birds arise spontaneously (see figure 1 for a compelling case study). Specific to this second point, an explosion [41] in mathematical models of cultural evolution means that it is possible to detect transmission processes, such as conformist transmission, underlying any such social learning of locally ‘colour-appropriate’ nest material, by analysing builder-birds' material-use patterns across the breeding season [42].

Admittedly, both of these hypothetical experiments are unrefined. In practice, replicate and counterbalanced (for nest-material colour) study groups would be required, as would controls for underlying genetic and/or arbitrary colour biases. The aim here, however, was not to provide polished experiments but to encourage their design. A possible study system is suggested in the following section.

## 4. One potential model ‘cultural’ system

As highlighted in a recent review [10], the zebra finch is becoming the *de facto* model system for empirical investigations of the socio-ecological dynamics underpinning avian nest construction, not least because they readily construct nests year-round under laboratory conditions (figure 2*c*). Critical to any role for material culture, laboratory experiments show that zebra finches use social information from both early and later life to guide their nest-construction decisions [27,43,44]; they will, for example, construct their first nest with the colour of material they had had access to as juveniles *if* an adult had also been present during that adolescent period [43]. In general, zebra finches are known to use social information in a wide range of contexts, including foraging decisions, predator detection, mate choice, and song learning [45]. Thus, their social learning coupled with their proven tractability as a study system, together also positions zebra finches as a powerful model to investigate cultural processes associated with nest construction, and animal material technology in general. That two of the cutting-edge tracking tools [33,38], detailed above, were streamlined in zebra finches (figure 2*a*), strengthens this suggestion, as does the documented flexibility to study the nest-construction behaviour of zebra finches in both laboratory and field conditions (recent empirical examples: [27,43,44,46–51]; figure 2*c*).

Because most birds build a nest to reproduce, there is, however, in theory, no shortage of potential model ‘cultural’ systems—an exciting prospect in terms of the scope for valuable [52] comparative research (across, for example, cooperative and non-cooperative breeders, migratory and non-migratory birds, and male and female builders). Such cross-species research need not be restricted to birds that will breed in nest-boxes; this point is compellingly illustrated in the backyard bird study by Williams [4] (and in figure 1). It is also worth noting that other species besides the zebra finch construct nests under captivity, including the near-ubiquitous house sparrow (*Passer domesticus*) [53,54] and the ‘renowned’ (for their hanging basket-like nests) village weaver (*Ploceus cucullatus*) [30,55,56]. Perhaps, however, it is the potential utility of the within-species variation in nest construction for phylogenetic methods that might appeal most, at least to cultural evolutionists. Specifically, it is conceivable that, in breeding bird populations under long-term study, and where their spatial genetic and ecological structures are known (e.g. [57]), phylogenetic tree-building methods could be used to test whether community variation in nest design can be accounted for, in part, by a cultural ‘signal’ [58], just as these methods have been successfully applied to similar questions regarding human-made artefacts such as baskets [59]. There is much scope for impactful inter- and intra-species comparative research into the interplay between life-history and behavioural variation, avian nest construction, and animal culture.

## 5. How avian nest construction progresses the experimental landscape

Aside from their logistic and comparative value, highlighted in the sections above, nest-building birds offer animal culture researchers at least one additional opportunity: to move beyond employing so-called ‘diffusion’ experiments, which typically document the spread (or lack thereof) of an introduced behaviour within and/or between animal groups [60–62]. Indeed, as they are reliant upon a single, or multiple subjects to demonstrate ‘what to do’, diffusion experiments typically require dedicated training blocks (occurring over days, weeks, and even months; e.g. [63–65]), and often additional time costs, such as finding (e.g. [66]), trapping (e.g. [67]) and/or releasing (e.g. [64]) suitable ‘models’, where suitability is in the eye of the experimenter(s). As such, traits like age, social rank, and general participation levels are frequent determinants of potential demonstrator quality (e.g. [63,66,68]), besides being able to consistently and correctly execute the trained behaviour. This self-selection process inevitably introduces sampling bias (or ‘STRANGE’ animals; [69]), which limits the extent that diffusion experiments can contribute meaningful inferences about purported real-world animal cultural phenomena. Avian nest construction, by contrast, requires neither training time commitments nor demonstrator selection; rather, builder-birds will simply get on with the ‘job’. In summary, although theoretical and empirical advances are being made, a fundamental understanding of the conditions under which culture in animals can present, propagate, and persist, particularly for naturally existing (that is, non-diffused) behaviours, is still in its infancy—nest-building birds could help to bridge this yawning gap.

## 6. Conclusion

For at least a century and a half, humans have observed human-like material culture in nest-building birds—but experimental support is still lacking. This knowledge deficit is true for the field of animal material culture as a whole, owing to ethical and logistical constraints associated with the long-standing main study systems of interest: tool making and/or using non-human primates and corvids. Avian nest construction, conversely, is a study system amenable to experimental manipulation in the laboratory and in the field; one that does not require training. Coupling this tractability with the diversity of tracking tools available, the time to include avian nest construction in animal culture research is now, if not long overdue.

## Data accessibility

This article does not contain any additional data.

## References

[1] Wallace AR. 1870 Contributions to the theory of natural selection. A series of essays. London, UK: Macmillan.

[2] Hochbaum HA. 1955 Travels and traditions of waterfowl. Minneapolis, MN: University of Minnesota Press.

[3] Mennerat A, Perret P, Lambrechts MM. 2009 Local individual preferences for nest materials in a passerine bird. PLoS ONE **4**, e5104. (10.1371/journal.pone.0005104) 19337365 PMC2659446

[4] Williams HS. 1934 Nest building-new style. Nat. Hist. **34**, 431-446.

[5] Schiermann G. 1939 ‘Stammesgenossenschaften’ bei Vögeln. Ornithol. Monatsbererichte **47**, 1-3.

[6] Aplin LM. 2019 Culture and cultural evolution in birds: a review of the evidence. Anim. Behav. **147**, 179-187. (10.1016/j.anbehav.2018.05.001)

[7] Aasen M, Slagsvold T. 2020 No cultural transmission of use of nest materials in titmice Paridae. Anim. Behav. **170**, 27-32. (10.1016/j.anbehav.2020.10.005)

[8] Bluff LA, Weir AA, Rutz C, Wimpenny JH, Kacelnik A. 2007 Tool-related cognition in New Caledonian crows. Comp. Cogn. Behav. Rev. **1**, 1-25. (10.3819/ccbr.2008.20001)

[9] Zentall TR. 2006 Mental time travel in animals: a challenging question. Behav. Process. **72**, 173-183. (10.1016/j.beproc.2006.01.009) 16466863

[10] Breen AJ, Guillette LM, Healy SD. 2016 What can nest-building birds teach us? Comp. Cogn. Behav. Rev. **11**, 83-102. (10.3819/ccbr.2016.110005)

[11] Whiten A. 2017 Culture extends the scope of evolutionary biology in the great apes. Proc. Natl Acad. Sci. USA **114**, 7790-7797. (10.1073/pnas.1620733114) 28739927 PMC5544264

[12] Rutz C, Hunt GR, St Clair JJH. 2018 Corvid technologies: how do New Caledonian crows get their tool designs? Curr. Biol. **28**, PR1109-PR1111. (10.1016/j.cub.2018.08.031) 30253153

[13] Rutz C, Hunt GR. 2020 New Caledonian crows afford invaluable comparative insights into human cumulative technological culture. Behav. Brain Sci. **43**, e177. (10.1017/S0140525X20000187) 32772983

[14] Fruth B, Tagg N, Stewart F. 2018 Sleep and nesting behavior in primates: a review. Am. J. Phys. Anthropol. **166**, 499-509. (10.1002/ajpa.23373) 29989164

[15] Laland KN, Janik VM. 2006 The animal cultures debate. Trends Ecol. Evol. **21**, 542-547. (10.1016/j.tree.2006.06.005) 16806574

[16] Caldwell CA, Millen AE. 2008 Studying cumulative cultural evolution in the laboratory. Phil. Trans. R. Soc. B **363**, 3529-3539. (10.1098/rstb.2008.0133) 18799419 PMC2607341

[17] Whiten A. 2021 The burgeoning reach of animal culture. Science **372**, eabe6514. (10.1126/science.abe6514) 33795431

[18] Tennie C, Call J, Tomasello M. 2009 Ratcheting up the ratchet: on the evolution of cumulative culture. Phil. Trans. R. Soc. B **364**, 2405-2415. (10.1098/rstb.2009.0052) 19620111 PMC2865079

[19] Laland KN. 2004 Social learning strategies. Learn. Behav. **32**, 4-14. (10.3758/BF03196002) 15161136

[20] Schlicht L, Valcu M, Kempenaers B. 2015 Male extraterritorial behavior predicts extrapair paternity pattern in blue tits, *Cyanistes caeruleus*. Behav. Ecol. **26**, 1404-1413. (10.1093/beheco/arv076)

[21] Järvinen P, Kluen E, Brommer JE. 2017 Low heritability of nest construction in a wild bird. Biol. Lett. **13**, 20170246. (10.1098/rsbl.2017.0246) 29046371 PMC5665766

[22] Whiten A. 2017 A second inheritance system: the extension of biology through culture. Interface Focus **7**, 20160142. (10.1098/rsfs.2016.0142) 28839918 PMC5566806

[23] Boesch C et al. 2020 Chimpanzee ethnography reveals unexpected cultural diversity. Nat. Hum. Behav. **4**, 910-916. (10.1038/s41562-020-0890-1) 32451479

[24] Camacho C, Canal D, Potti J. 2016 Natal habitat imprinting counteracts the diversifying effects of phenotype-dependent dispersal in a spatially structured population. BMC Evol. Biol. **16**, 158-167. (10.1186/s12862-016-0724-y) 27503506 PMC4976508

[25] Smith JE, Pinter-Wollman N. 2021 Observing the unwatchable: integrating automated sensing, naturalistic observations and animal social network analysis in the age of big data. J. Anim. Ecol. **90**, 62-75. (10.1111/1365-2656.13362) 33020914

[26] Krause J, Krause S, Arlinghaus R, Psorakis I, Roberts S, Rutz C. 2013 Reality mining of animal social systems. Trends Ecol. Evol. **28**, 541-551. (10.1016/j.tree.2013.06.002) 23856617

[27] Guillette LM, Scott ACY, Healy SD. 2016 Social learning in nest-building birds: a role for familiarity. Proc. R. Soc. B **283**, 20152685. (10.1098/rspb.2015.2685) PMC4822453 27009230

[28] Coussi-Korbel S, Fragaszy DM. 1995 On the relation between social dynamics and social learning. Anim. Behav. **50**, 1441-1453. (10.1016/0003-3472(95)80001-8)

[29] Kendal RL, Boogert NJ, Rendell L, Laland KN, Webster M, Jones PL. 2018 Social learning strategies: bridge-building between fields. Trends Cogn. Sci. **22**, 651-665. (10.1016/j.tics.2018.04.003) 29759889

[30] Collias EC, Collias NE. 1973 Further studies on development of nest-building behaviour in a weaverbird (*Ploceus cucullatus*). Anim. Behav. **21**, 371-382. (10.1016/S0003-3472(73)80079-X)

[31] Hasenjager MJ, Leadbeater E, Hoppitt W. 2021 Detecting and quantifying social transmission using network-based diffusion analysis. J. Anim. Ecol. **90**, 8-26. (10.1111/1365-2656.13307) 32745269

[32] Maldonado-Chaparro AA, Alarcón-Nieto G, Klarevas-Irby JA, Farine DR. 2018 Experimental disturbances reveal group-level costs of social instability. Proc. R. Soc. B **285**, 20181577. (10.1098/rspb.2018.1577) PMC6253379 30429300

[33] Alarcón-Nieto G, Graving JM, Klarevas-Irby JA, Maldonado-Chaparro AA, Mueller I, Farine DR. 2018 An automated barcode tracking system for behavioural studies in birds. Methods Ecol. Evol. **9**, 1536-1547. (10.1111/2041-210X.13005)

[34] Ogino M, Maldonado-Chaparro AA, Farine DR. 2021 Drivers of alloparental provisioning of fledglings in a colonially breeding bird. Behav. Ecol. **32**, 316-326. (10.1093/beheco/araa137)

[35] Aplin LM, Sheldon BC, McElreath R. 2017 Conformity does not perpetuate suboptimal traditions in a wild population of songbirds. Proc. Natl Acad. Sci. USA **114**, 7830-7837. (10.1073/pnas.1621067114) 28739943 PMC5544276

[36] Hillemann F, Cole EF, Keen SC, Sheldon BC, Farine DR. 2019 Diurnal variation in the production of vocal information about food supports a model of social adjustment in wild songbirds. Proc. R. Soc. B **286**, 20182740. (10.1098/rspb.2018.2740) PMC6408885 30963842

[37] Hillemann F, Cole EF, Sheldon BC, Farine DR. 2020 Information use in foraging flocks of songbirds: no evidence for social transmission of patch quality. Anim. Behav. **165**, 35-41. (10.1016/j.anbehav.2020.04.024)

[38] Ferreira AC, Silva LR, Renna F, Brandl HB, Renoult JP, Farine DR, Covas R, Doutrelant C. 2020 Deep learning-based methods for individual recognition in small birds. Methods Ecol. Evol. **11**, 1072-1085. (10.1111/2041-210X.13436)

[39] Mainwaring MC, Hartley IR, Lambrechts MM, Deeming DC. 2014 The design and function of birds' nests. Ecol. Evol. **4**, 3909-3928. (10.1002/ece3.1054) 25505520 PMC4242575

[40] Surgery J, Du Feu CR, Deeming CD. 2012 Opportunistic use of a wool-like artificial material as lining of tit (*Paridae*) nests. Condor **114**, 385-392. (10.1525/cond.2012.110111)

[41] Mesoudi A. 2016 Cultural evolution: a review of theory, findings and controversies. Evol. Biol. **43**, 481-497. (10.1007/s11692-015-9320-0)

[42] Kandler A, Powell A. 2015 Inferring learning strategies from cultural frequency data. In Learning strategies and cultural evolution during the palaeolithic (eds A Mesoudi, K Aoki), pp. 85-101. Tokyo, Japan: Springer.

[43] Breen AJ, Lovie KE, Guerard C, Edwards SC, Cooper J, Healy SD, Guillette LM. 2020 Juvenile socio-ecological environment shapes material technology in nest-building birds. Behav. Ecol. **31**, 892-901. (10.1093/beheco/araa027)

[44] Breen AJ, Bonneaud CC, Healy SD, Guillette LM. 2019 Social learning about construction behaviour via an artefact. Anim. Cogn. **22**, 305-315. (10.1007/s10071-019-01240-x) 30767145 PMC6507502

[45] Zann R. 1996 The zebra finch: a synthesis of laboratory and field studies. New York, NY: Oxford University Press.

[46] Edwards SC, Shoot TT, Martin RJ, Sherry DF, Healy SD. 2020 It's not all about temperature: breeding success also affects nest design. Behav. Ecol. **31**, 1065-1072. (10.1093/beheco/araa052)

[47] Camacho-Alpízar A, Eckersley T, Lambert CT, Balasubramanian G, Guillette LM. 2021 If it ain't broke don't fix it: breeding success affects nest-building decisions. Behav. Process. **184**, 104336. (10.1016/j.beproc.2021.104336) 33513432

[48] Campbell BL, Hurley LL, Griffith SC. 2018 Behavioural plasticity under a changing climate; how an experimental local climate affects the nest construction of the zebra finch *Taeniopygia guttata*. J. Avian Biol. **49**, jav-01717. (10.1111/njb.01717)

[49] Brandl HB, Griffith SC, Schuett W. 2018 Wild zebra finches do not use social information from conspecific reproductive success for nest site choice and clutch size decisions. Behav. Ecol. Sociobiol. **72**, 1-11. (10.1007/s00265-018-2533-3)

[50] Brandl HB, Griffith SC, Farine DR, Schuett W. 2021 Wild zebra finches that nest synchronously have long-term stable social ties. J. Anim. Ecol. **90**, 76-86. (10.1111/1365-2656.13082) 31407336

[51] Brandl HB, Griffith SC, Schuett W. 2019 Wild zebra finches choose neighbours for synchronized breeding. Anim. Behav. **151**, 21-28. (10.1016/j.anbehav.2019.03.002)

[52] Breen AJ, Sugasawa S, Healy SD. 2021 Manipulative and technological skills do not require a slow life history. Front. Ecol. Evol. **9**, 635802. (10.3389/fevo.2021.635802)

[53] Mitchell CJ, Hayes RO. 1973 Breeding house sparrows, *Passer domesticus* in captivity. Ornithol. Monogr. **14**, 39-48. (10.2307/40168056)

[54] Girndt A, Cockburn G, Sánchez-Tójar A, Hertel M, Burke T, Schroeder J. 2019 Male age and its association with reproductive traits in captive and wild house sparrows. J. Evol. Biol. **32**, 1432-1443. (10.1111/jeb.13542) 31529748 PMC8653889

[55] Collias EC, Collias NE. 1964 The development of nest-building behavior in a weaverbird. Auk **81**, 42-52. (10.2307/4082609)

[56] Collias NE, Collias EC. 1962 An experimental study of the mechanisms of nest building in a weaverbird. Auk **79**, 568-595. (10.2307/4082640)

[57] Harrison XA, York JE, Young AJ. 2014 Population genetic structure and direct observations reveal sex-reversed patterns of dispersal in a cooperative bird. Mol. Ecol. **23**, 5740-5755. (10.1111/mec.12978) 25346189 PMC4265262

[58] Mace R, Holden CJ. 2005 A phylogenetic approach to cultural evolution. Trends Ecol. Evol. **20**, 116-121. (10.1016/j.tree.2004.12.002) 16701354

[59] Jordan P, Shennan S. 2003 Cultural transmission, language, and basketry traditions amongst the California Indians. J. Anthropol. Archaeol. **22**, 42-74. (10.1016/S0278-4165(03)00004-7)

[60] Whiten A, Mesoudi A. 2008 Establishing an experimental science of culture: animal social diffusion experiments. Phil. Trans. R. Soc. B **363**, 3477-3488. (10.1098/rstb.2008.0134) 18799418 PMC2607342

[61] Duboscq J, Romano V, MacIntosh A, Sueur C. 2016 Social information transmission in animals: lessons from studies of diffusion. Front. Psychol. **7**, 1147. (10.3389/fpsyg.2016.01147) 27540368 PMC4973104

[62] Whiten A, Caldwell CA, Mesoudi A. 2016 Cultural diffusion in humans and other animals. Curr. Opin. Psychol. **8**, 15-21. (10.1016/j.copsyc.2015.09.002) 29506791

[63] Alem S, Perry CJ, Zhu X, Loukola OJ, Ingraham T, Søvik E, Chittka L. 2016 Associative mechanisms allow for social learning and cultural transmission of string pulling in an insect. PLoS Biol. **14**, e1002564. (10.1371/journal.pbio.1002564) 27701411 PMC5049772

[64] Aplin LM, Farine DR, Morand-Ferron J, Cockburn A, Thornton A, Sheldon BC. 2015 Experimentally induced innovations lead to persistent culture via conformity in wild birds. Nature **518**, 538-541. (10.1038/nature13998) 25470065 PMC4344839

[65] van de Waal E, Borgeaud C, Whiten A. 2013 Potent social learning and conformity shape a wild primate's foraging decisions. Science **340**, 483-485. (10.1126/science.1232769) 23620053

[66] Thornton A, Malapert A. 2009 The rise and fall of an arbitrary tradition: an experiment with wild meerkats. Proc. R. Soc. B **276**, 1269-1276. (10.1098/rspb.2008.1794) PMC2660974 19141416

[67] Curio E, Ernst U, Vieth W. 1978 The adaptive significance of avian mobbing. Z. Tierpsychol. **48**, 184-202. (10.1111/j.1439-0310.1978.tb00255.x)

[68] Whiten A, Horner V, De Waal FBM. 2005 Conformity to cultural norms of tool use in chimpanzees. Nature **437**, 737-740. (10.1038/nature04047) 16113685

[69] Webster MM, Rutz C. 2020 How STRANGE are your study animals? Nature **582**, 337-340. (10.1038/d41586-020-01751-5) 32541916

